# Delayed Potassium Application Alters Protein Accumulation While Improving Eating Quality and Yield in Rice

**DOI:** 10.3390/plants15030437

**Published:** 2026-01-30

**Authors:** Mingming Hu, Chaoyue Wu, Guangyi Chen, Tian Li

**Affiliations:** 1 Crop Ecophysiology and Cultivation Key Laboratory of Sichuan Province, College of Agronomy, Sichuan Agricultural University, Chengdu 611130, China; 2020201029@stu.sicau.edu.cn (M.H.);; 2 Rice Research Institute, Sichuan Agricultural University, Chengdu 611130, China

**Keywords:** rice, delayed potassium fertilizer application, protein synthesis, eating quality

## Abstract

Potassium plays a critical role in improving rice yield and quality; however, the effects of delayed K application on grain protein metabolism and eating quality remain unclear. This study evaluated four potassium fertilizer management modes designed with basal fertilizer: panicle fertilizer, with ratios of 10:0 (K1, control), 8:2 (K2), 6:4 (K3), and 4:6 (K4). Grain protein and protein fractions, free amino acids (FAA), key protein synthesis enzyme activities, starch RVA profile, taste value, processing and appearance quality, and yield were measured. With the delayed application of potassium fertilizer, grain GS, and GOGAT activities decreased by 3.15–7.67% and 6.92–13.06%, while GOT and GPT activities declined by 3.72–13.99% and 2.94–3.32% compared with K1. Consequently, FAA, albumin, globulin, prolamin, and glutelin contents all decreased, and grain protein content was significantly reduced, by 0.18–0.44 percentage points. In contrast, peak viscosity and breakdown increased, setback decreased, and taste value improved by 4.35–10.14%. Meanwhile, head rice rate increased, chalky grain rate and chalkiness decreased, and yield under K4 treatment increased significantly by 3.48%. Delayed potassium fertilizer application (K4 treatment) helps to reduce the activity of key enzymes involved in protein synthesis, decrease protein and its component contents, while simultaneously improving yield and eating quality.

## 1. Introduction

Rice is the staple food for more than 65% of China’s population and is the most important food crop in the national diet [1,2]. With the upgrading of consumption, the demand for rice has shifted from a sole focus on yield to a balance between both quality and yield, with high-quality rice increasingly favored in the market [3,4]. Among these, eating quality is the core indicator for evaluating staple rice, as it directly determines the texture and palatability of rice, making it the most concerned quality characteristic for consumers [5].

Protein is the second-largest storage substance in rice endosperm after starch and plays an important role in eating quality [6]. Previous studies have shown that excessively high protein content can inhibit starch absorption, expansion, and gelatinization, thus lowering the eating quality of rice [7,8]. Moreover, the relationship between protein content and eating quality is not simply linear but exhibits a threshold effect—a moderate reduction in protein content can improve eating quality, whereas excessively high or low levels both result in quality deterioration [9]. At the level of protein fractions, gliadin has been reported to hinder starch structure formation and is difficult to digest, thus markedly reducing eating quality, while albumin, globulin, and glutelin, which are nutritionally valuable, generally do not exert negative effects [10]. However, some studies have suggested that all four protein fractions may adversely affect rice eating quality [11]. Taken together, these findings indicate that the relationship between protein fractions and rice eating quality remains inconclusive. Therefore, clarifying the effects of protein and its fractions on eating quality, and exploring cultivation strategies to regulate protein synthesis metabolism, is of great significance for the breeding and production of high-quality rice.

Potassium is an essential element for rice growth and development; it is extensively involved in physiological and metabolic processes, including enzyme activation, osmotic regulation, and nutrient transport [12]. It plays an irreplaceable role in the yield formation, quality improvement, and stress resistance of crops [13]. At the molecular level, K^+^ can regulate the translation process of mRNA to maintain coordination between tRNA and ribosomes, thereby ensuring the normal progression of protein synthesis [14]. In addition, K helps to maintain intracellular charge balance and osmotic stability, providing a favorable ionic environment for metabolic activities [15]. Importantly, many enzymes associated with protein synthesis, such as glutamine synthetase (GS), glutamate synthase (GOGAT), and aminotransferases (GOT, GPT), are K-dependent or strongly influenced by cellular K levels [16]. Potassium ions not only stabilize enzyme conformation to enhance catalytic efficiency but also support energy metabolism and provide substrates for synthesis, thereby promoting amino acid biosynthesis and transport, which in turn regulates protein accumulation and composition in rice grains [17]. Conversely, K deficiency suppresses the activity of related enzymes, alters nitrogen assimilation pathways, and ultimately affects protein accumulation and quality formation in rice grains.

In the southern rice-growing regions of China, more than 50% of farmland suffers from varying degrees of potassium (K) deficiency; the depletion of soil K continues to worsen, severely restricting improvements in rice yield and quality [18]. Potassium is readily adsorbed by soil exchange sites, particularly in potassium-deficient soils, which may reduce its immediate availability to crops [19]. However, rice exhibits a distinct temporal pattern of potassium demand, with rapid uptake occurring during panicle development and early grain-filling. Split potassium application has, therefore, been proposed as an effective strategy with which to synchronize potassium supply with plant demand [20]. Basal potassium fertilization helps to replenish exchangeable potassium pools, while a delayed application during panicle development shortens the soil residence time of potassium prior to uptake, potentially reducing fixation losses and enhancing root absorption efficiency [21]. This strategy may be particularly relevant in potassium-deficient soils, where improving the timing of potassium availability could be as important as increasing total potassium input.

Previous studies have shown that rational K fertilization can effectively enhance photosynthetic capacity, promote nitrogen absorption and translocation, regulate amino acid and protein synthesis, and ultimately increase yield [22,23]. On the other hand, sufficient K supply promotes the formation and translocation of photosynthetic products, thereby facilitating sucrose and starch accumulation in grains and improving the carbon–nitrogen metabolic cycle [24]. In addition, K fertilization has been reported to reduce chalky grain rate, chalkiness, and amylose content, while improving milling quality, gel consistency, and protein content, thereby enhancing eating quality [25,26]. However, most existing studies have focused on yield and general quality traits, whereas systematic investigations on how different K fertilizer management modes during the grain-filling stage regulate protein synthesis metabolism and how these changes affect eating quality are still lacking.

Therefore, this study aims to investigate the regulatory effects of delayed potassium fertilizer application on protein synthesis metabolism and eating quality in rice grains. The findings will elucidate the physiological mechanisms by which potassium regulates grain protein synthesis and quality formation, thereby providing a theoretical basis for the production of high-quality rice.

## 2. Materials and Methods

### 2.1. Experimental Site and Materials

The experiment was conducted from 2019 to 2021 in Huihe Village, Wenjiang District, Chengdu City, Sichuan Province (30°43′ N, 103°47′ E), with the previous crop being rapeseed. The soil texture in the plow layer (0–20 cm) was sandy loam, and the tested variety was the hybrid indica rice Yixiangyou 2115, which is widely cultivated in the southwestern rice-growing region. The basic chemical properties of the soil are shown in Table 1. Soil pH, organic matter, total nitrogen, available phosphorus, and available potassium were determined following standard soil analysis procedures described by Bao [27]. The basic soil test showed that the available K content in the experimental area was only 50~57 mg kg^−1^, which is considered a deficient level for paddy soils in southwest China.

### 2.2. Experimental Design

A single-factor randomized complete block design was employed to evaluate the effects of potassium fertilizer timing. Four potassium fertilizer management modes were established based on different proportions of basal and panicle-stage potassium application, with a constant total potassium rate of 180 kg K_2_O ha^−1^ across all treatments, consistent with local high-yield cultivation practices. The potassium treatments were defined as follows: K1 (control): 100% of potassium applied as basal fertilizer before transplanting (basal:panicle = 10:0); K2: 80% of potassium applied as basal fertilizer and 20% applied at the panicle initiation stage (basal:panicle = 8:2); K3: 60% of potassium applied as basal fertilizer and 40% applied at the panicle initiation stage (basal:panicle = 6:4); K4: 40% of potassium applied as basal fertilizer and 60% applied at the panicle initiation stage (basal:panicle = 4:6). K was applied as KCl (K_2_O ≥ 60%); therefore, chloride was co-applied and varied in timing together with K. Basal potassium fertilizer was uniformly broadcast and incorporated into the soil one day before transplanting, whereas panicle-stage potassium fertilizer was top-dressed during early panicle differentiation. Nitrogen fertilizer was applied as urea (N ≥ 46%) at a rate of 180 kg N ha^−1^, split between basal and tillering stages, at a ratio of 6:4. Basal nitrogen fertilizer was applied together with basal potassium fertilizer prior to transplanting; tillering nitrogen fertilizer was applied 7 days after transplanting. Phosphorus fertilizer was applied as calcium superphosphate (P_2_O_5_ ≥ 12%) at 90 kg ha^−1^ and was entirely applied as basal fertilizer. For all treatments, basal nitrogen, basal potassium, and phosphorus fertilizers were applied one day before transplanting. The second potassium application was applied at the fourth leaf stage from the flag leaf (i.e., the N−4 leaf stage). Other agronomic practices, including irrigation, pest control, and weed management, followed local standard cultivation practices. Each plot measured 25 m^2^ (5 m × 5 m), and each treatment was replicated three times, resulting in a total of 12 plots. Plots were separated by 0.5 m-wide ridges, which were covered with plastic film to prevent water and fertilizer movement between plots.

### 2.3. Measurements

#### 2.3.1. Sample Collection

Healthy rice panicles with consistent growth and at the same heading stage were selected and marked during the heading stage. Starting from 10 days after flowering, rice panicles from each treatment group were sampled every 5 days (a total of 6 times). For each sampling, 40 panicles were collected, half of which were treated with liquid nitrogen and stored at −80 °C in a deep freezer for measuring key enzyme activities involved in protein synthesis in the grains. The other half was subjected to a killing process at 105 °C for 30 min, then dried at 80 °C until a constant weight was reached. After grinding and sieving, the samples were used to measure protein, protein components, and free amino acid content.

#### 2.3.2. Grain Protein and Protein Component Content

Grain protein content was determined using the Kjeldahl method. Ground brown rice samples (1.00 g) were digested with concentrated sulfuric acid in the presence of potassium sulfate and copper sulfate as catalysts at 420 °C for 1 h. After digestion, the released ammonia was distilled and absorbed in a boric acid solution (40 g L^−1^) containing mixed indicators, and nitrogen content was determined using an automatic Kjeldahl nitrogen analyzer (Kjeltec 2300, FOSS, Hillerød, Denmark). Protein content was calculated by multiplying the nitrogen content by a conversion factor of 5.95 [28].

Protein fractions were sequentially extracted according to the Osborne fractionation principle, which separates proteins based on their differential solubility in water, salt solution, alcohol, and dilute alkali. Brown rice flour samples (0.30 g) were extracted successively with ultrapure water, 0.6 mol L^−1^ NaCl, 80% (*v*/*v*) ethanol, and 0.06 mol L^−1^ NaOH to obtain albumin, globulin, prolamin, and glutelin fractions, respectively. This extraction order has been widely applied and validated for rice grain protein analysis [29]. The concentration of each protein fraction was determined using the Bradford assay, with absorbance measured at 595 nm. Protein contents were calculated based on standard curves prepared using bovine serum albumin (BSA) as the calibration standard.

#### 2.3.3. Grain Free Amino Acid Content

Free amino acids were extracted from ground rice grain samples using aqueous extraction followed by protein precipitation [11]. Briefly, powdered samples (0.4 g) were extracted with ultrapure water under low-temperature shaking conditions. After extraction, samples were heat-treated to inactivate enzymes and centrifuged to obtain the supernatant. Proteins were precipitated by adding sulfosalicylic acid (10%, *w*/*v*) and the resulting supernatant was collected after centrifugation. Free amino acid concentrations were subsequently determined using an automatic amino acid analyzer (A300, MembraPure, Hennigsdorf, Germany).

#### 2.3.4. Key Enzyme Activities in Grain Protein Synthesis

A total of 2.00 g of dehulled rice grains collected from the middle portion of the panicle was used for enzyme analysis. The activities of key protein synthesis-related enzymes, including glutamine synthetase (GS), glutamate synthase (GOGAT), glutamate oxaloacetate transaminase (GOT), and glutamate pyruvate transaminase (GPT), were determined using enzyme-linked immunosorbent assay (ELISA) kits (Shanghai Fankel Industrial Co., Ltd., Shanghai, China), which have been validated for plant tissues, including rice grains, and have been widely applied in nitrogen metabolism studies [30].

Crude enzyme extracts were prepared by homogenizing grain samples in phosphate-buffered saline (PBS, pH 7.2–7.4) under ice-cold conditions, followed by centrifugation at low temperature (2–8 °C). The resulting supernatants were used for ELISA analysis. Briefly, enzyme standards and sample extracts were incubated in antibody-coated microplate wells, followed by reaction with horseradish peroxidase (HRP)-conjugated detection antibodies. After washing to remove unbound components, chromogenic substrates were added to initiate color development. The reaction was terminated using a stop solution; absorbance was measured at 450 nm using a microplate reader. The enzyme activities of GS, GOGAT, GOT, and GPT were calculated based on standard curves, according to the manufacturer’s instructions.

#### 2.3.5. Starch RVA Profile

The starch pasting properties of rice flour were determined using a Rapid Visco Analyzer (RVA-4, Newport Scientific, Warriewood, Australia) [28]. Rice noodles were sieved through a 0.15 mm sieve; 3.00 g of brown rice was weighed for each sample; and 25.00 mL distilled water was added. The measurement time was 12.5 min. During this process, the temperature inside the tank was first maintained at 50 °C for 1 min, then rose to 95 °C for 2.5 min, and finally dropped to 50 °C for 1.4 min. The temperature change rate was 11.84 °C min^−1^. The speed of the mixer was 960 r min^−1^ for the first 10 s and then maintained at 160 r min^−1^. Data acquisition and analysis were performed using Thermal Cycle for Windows (TCW) software. The parameters recorded included peak viscosity, hot paste viscosity, breakdown, final viscosity, setback, and gelatinization temperature. All viscosity values were expressed in rapid Visco units (RVU).

#### 2.3.6. Taste Value

The taste quality of the cooked rice was evaluated using a rice taste analyzer (STA 1B, Satake Asia Co., Ltd., Tokyo, Japan) [28]. Polished rice samples were prepared under standardized cooking conditions. Briefly, rice was soaked, rinsed, and steamed with distilled water at a rice-to-water ratio of 1:1.4. After cooking, samples were allowed to equilibrate under controlled cooling conditions prior to analysis. The cooked rice was then molded into uniform rice cakes; taste value, appearance, and texture parameters were determined using the rice taste analyzer. Rice hardness, viscosity, and balance were further measured using a rice hardness/viscosity meter (RHS 1A, Satake Asia Co., Ltd., Tokyo, Japan).

#### 2.3.7. Processing and Appearance Quality

The brown rice rate, milled rice rate, headed rice rate, chalky grain rate, chalkiness, and length–width ratio of the rice were determined according to the national standard of the People’s Republic of China, GB/T 17891-2017 High quality rice [28].

#### 2.3.8. Yield and Yield Composition Factors

At the rice maturity stage, 60 random plants from each treatment were selected to investigate the effective panicle number. For each plot, 5 representative plants were chosen and the panicles were clipped and placed in envelopes for further examination. The key indicators for seed examination included: the number of filled grains, unfilled grains, seed-setting rate, and 1000-grain weight. Additionally, each plot was harvested separately, and the actual yield was calculated based on a moisture content of 13.5% for the paddy rice.

### 2.4. Statistical Analysis

Data were organized using Microsoft Excel 2016 (Microsoft Corp., Redmond, WA, USA), and statistical analysis was performed in SPSS 27.0 (IBM Corp., Armonk, NY, USA). Comparisons were made using the least significant difference (LSD) method at a significance level of *p* < 0.05. Graphs were plotted using Origin 2024 (OriginLab, Northampton, MA, USA).

## 3. Results

### 3.1. Protein Content

**Figure 1:**
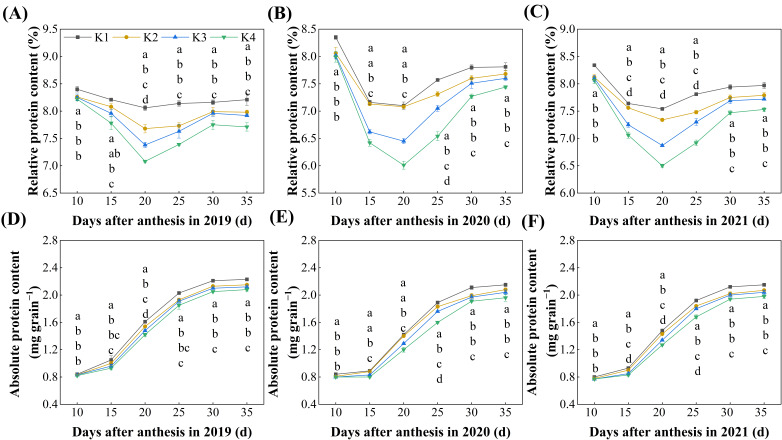
Effect of delayed potassium fertilizer application on rice protein content. Different lowercase letters on the same day mean the significant difference between treatments at *p* < 0.05. “Relative protein content” refers to the proportion of protein in the total grain dry matter, expressed as a percentage (%).(**A**) Changes in relative protein content during grain filling in 2019; (**B**) changes in relative protein content during grain filling in 2020; (**C**) changes in relative protein content during grain filling in 2021; (**D**) changes in absolute protein content during grain filling in 2019; (**E**) changes in absolute protein content during grain filling in 2020; (**F**) changes in absolute protein content during grain filling in 2021.

Delayed potassium fertilizer application significantly affected the accumulation dynamics of protein content during the grain-filling period (Figure 1). As the grain-filling progressed, the relative protein content of the grains showed a trend of first decreasing and then increasing, reaching the lowest value 20 days after flowering, and gradually rising thereafter. With the delayed application of potassium fertilizer, the relative protein content of each treatment followed the order: K1 > K2 > K3 > K4, with significant differences observed between 20~35 days after flowering. At 35 days after flowering (maturity), the relative protein content of the grains in the delayed potassium fertilizer treatments was significantly lower—by 0.18–0.44 percentage points—compared to K1; no significant difference was found between K2 and K3 treatments (Figure 1A–C).

The absolute protein content continued to increase throughout the grain-filling period, stabilizing between 30~35 days. With the delayed potassium fertilizer application, the absolute protein content in each treatment showed a declining trend. At 35 days after flowering (maturity), the absolute protein content in the delayed potassium fertilizer treatments was significantly lower by 3.32~7.66% compared to K1; however, no significant difference was observed between K2 and K3 treatments (Figure 1D–F).

### 3.2. Protein Component Content

**Figure 2:**
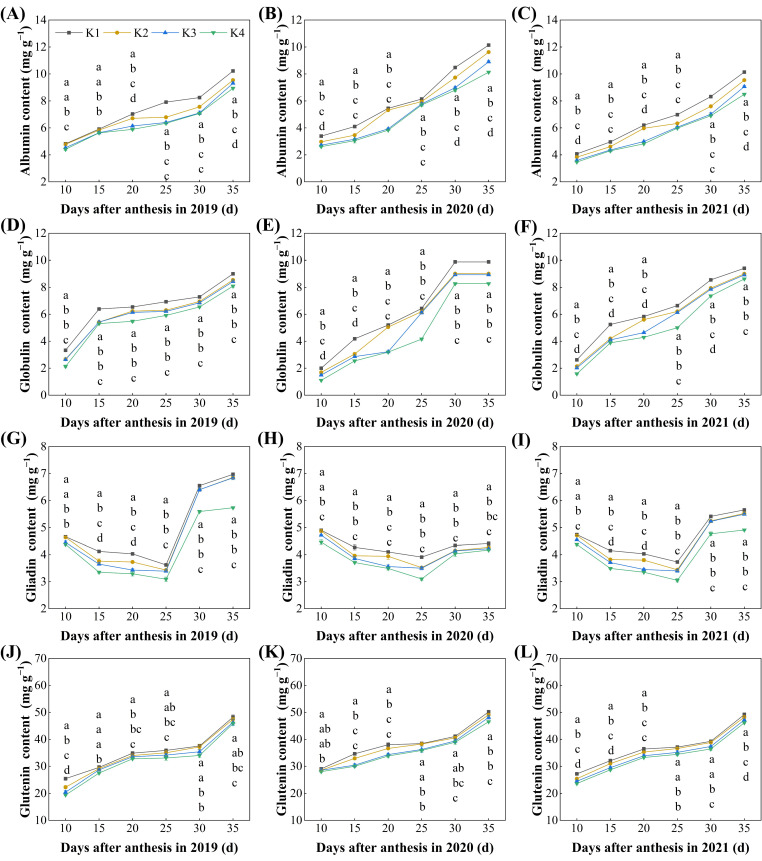
Effect of delayed potassium fertilizer application on rice protein component content. Different lowercase letters on the same day mean the significant difference between treatments at *p* < 0.05. (**A**) Changes in albumin content during grain filling in 2019; (**B**) changes in albumin content during grain filling in 2020; (**C**) changes in albumin content during grain filling in 2021; (**D**) changes in globulin content during grain filling in 2019; (**E**) changes in globulin content during grain filling in 2020; (**F**) changes in globulin content during grain filling in 2021; (**G**) changes in gliadin content during grain filling in 2019; (**H**) changes in gliadin content during grain filling in 2020; (**I**) changes in gliadin content during grain filling in 2021; (**J**) changes in glutenin content during grain filling in 2019; (**K**) changes in glutenin content during grain filling in 2020; (**L**) changes in glutenin content during grain filling in 2021.

Delayed potassium fertilizer application significantly affected the accumulation dynamics of the four protein components during the grain-filling period (Figure 2). As the grain-filling progressed, the content of albumin and globulin in the grains showed a steady increase, with the K1 treatment consistently the highest, K4 the lowest, and K2 and K3 decreasing sequentially. Significant differences were observed between 20~35 days after flowering. At 35 days after flowering (maturity), the albumin and globulin content in the grains from the delayed potassium fertilizer treatments were significantly lower than K1 by 5.85~16.18% and 4.37~8.30%, respectively. However, no significant difference in globulin content was observed between K2 and K3 treatments (Figure 2A–F).

The content of gliadin protein exhibited a trend of first decreasing and then increasing, reaching the lowest value 25 days after flowering, and gradually rising afterward, following the order of K1 > K2 > K3 > K4. At 35 days after flowering (maturity), the gliadin protein content in the grains from the delayed potassium fertilizer treatments was significantly lower than K1, by 2.33~13.03% (Figure 2H–J). The glutenin content continuously increased and made the largest contribution to total protein accumulation, also following the order of K1 as the highest and K4 the lowest. At 35 days after flowering (maturity), the glutenin content in the grains from the delayed potassium fertilizer treatments was significantly lower than K1, by 1.93~6.20% (Figure 2K–M).

### 3.3. Proportion of Protein Components

**Figure 3:**
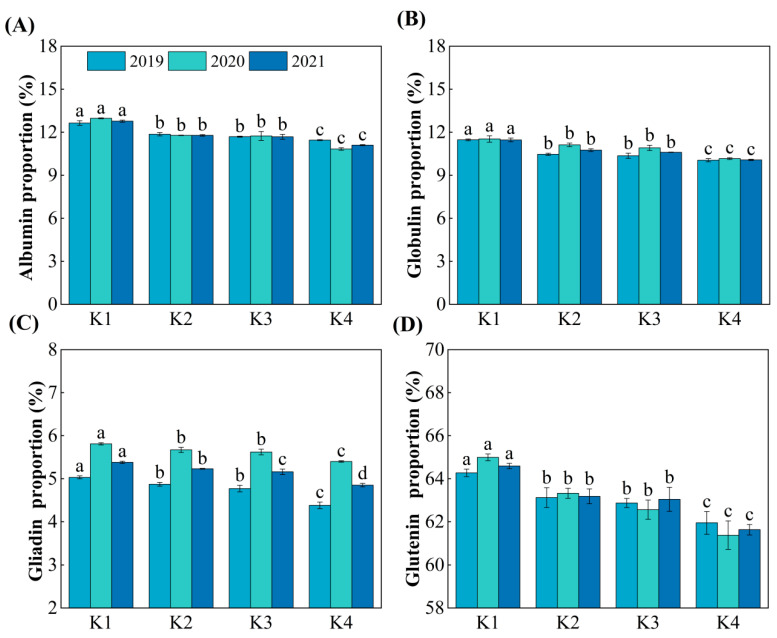
Effect of delayed potassium fertilizer application on the proportion of rice protein component. Different lowercase letters indicate significant differences among treatments within the same year at *p* < 0.05. (**A**) Proportion of albumin under different potassium treatments in 2019–2021; (**B**) proportion of globulin under different potassium treatments in 2019–2021; (**C**) proportion of gliadin under different potassium treatments in 2019–2021; (**D**) proportion of glutenin under different potassium treatments in 2019–2021.

Delayed potassium fertilizer application significantly affected the proportion of the four protein components in grains at maturity (Figure 3). With the delayed application of potassium fertilizer, the proportion of each protein component followed the order of K1 > K2 > K3 > K4. The content of albumin and globulin in grains from the delayed potassium fertilizer treatments was significantly reduced, by 0.99–1.67 percentage points and 0.71–1.39 percentage points, respectively, compared to K1 (Figure 3A,B). The content of gliadin and glutelin was significantly reduced, by 0.15–0.53 percentage points and 1.41–2.96 percentage points, respectively, compared to K1 (Figure 3C,D). Additionally, there was no significant difference in the proportion of the four protein components between K2 and K3 treatments.

### 3.4. Free Amino Acid Content

**Figure 4:**
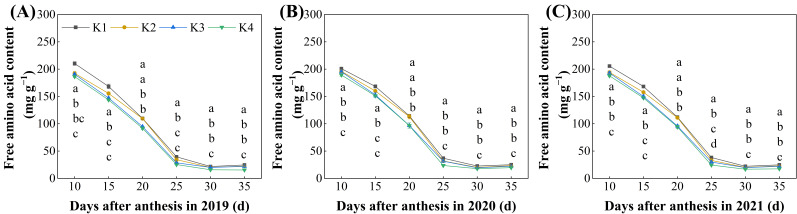
Effect of delayed potassium fertilizer application on rice free amino acid content. Different lowercase letters on the same day mean the significant difference between treatments at *p* < 0.05. (**A**) Changes in free amino acid content during grain filling in 2019; (**B**) changes in free amino acid content during grain filling in 2020; (**C**) changes in free amino acid content during grain filling in 2021.

Delayed potassium fertilizer application significantly affected the accumulation dynamics of free amino acids during the grain-filling period (Figure 4). As an important precursor in protein synthesis, the free amino acid content decreased as the grain-filling progressed, with the most rapid decline occurring between 10~25 days after flowering; the levels stabilized between 25~35 days after flowering. With the delayed application of potassium fertilizer, the free amino acid content followed the order of K1 > K2 > K3 > K4. At 35 days after flowering (maturity), the free amino acid content in grains from the delayed potassium fertilizer treatments was significantly reduced, by 8.01~29.59%, compared to K1, although no significant difference was found between K2 and K3 treatments (Figure 4A–C).

### 3.5. Key Enzyme Activities in Grain Protein Synthesis

**Figure 5:**
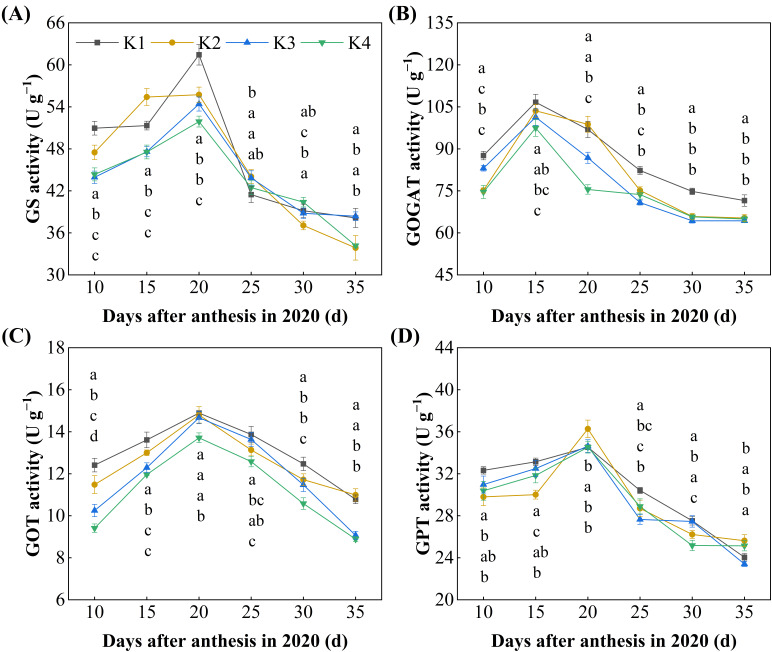
Effect of delayed potassium fertilizer application on rice key enzyme activity in protein synthesis. Different lowercase letters on the same day mean the significant difference between treatments at *p* < 0.05. (**A**) Changes in glutamine synthetase (GS) activity during grain filling; (**B**) changes in glutamate synthase (GOGAT) activity during grain filling; (**C**) changes in glutamate oxaloacetate transaminase (GOT) activity during grain filling; (**D**) changes in glutamate pyruvate transaminase (GPT) activity during grain filling.

Delayed potassium fertilizer application significantly affected the dynamic of key enzyme activities in protein synthesis during the grain-filling period (Figure 5). As the filling process progressed, the activity of GS, GOT, and GPT all peaked at 20 days after flowering, while GOGAT activity peaked at 15 days after flowering. With the delayed application of potassium fertilizer, the average enzyme activities in grains followed the order of K1 > K2 > K3 > K4. The activity of GS and GOGAT in the delayed potassium fertilizer treatments was reduced by 3.15~7.67% and 6.92~13.06%, respectively, compared to K1 (Figure 5A,B). Similarly, the activity of GOT and GPT was reduced by 3.72~13.99% and 2.94~3.32%, respectively, compared to K1 (Figure 5C,D).

### 3.6. Starch RVA Profile

**Figure 6:**
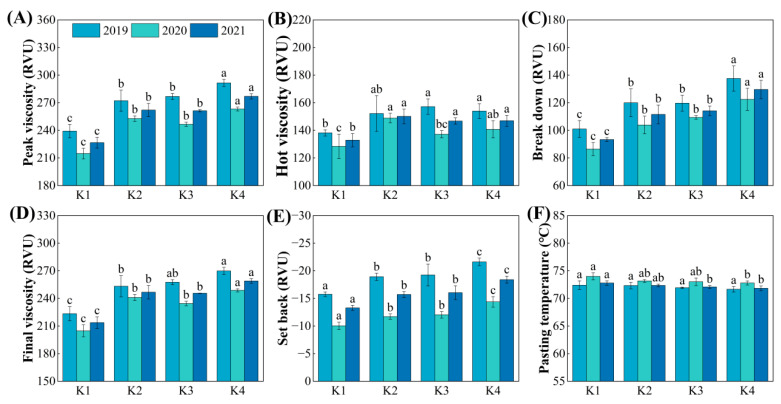
Effect of delayed potassium fertilizer application on rice starch RVA profile. Different lowercase letters indicate significant differences among treatments within the same year at *p* < 0.05. (**A**) Peak viscosity under different potassium treatments in 2019–2021; (**B**) hot viscosity under different potassium treatments in 2019–2021; (**C**) breakdown under different potassium treatments in 2019–2021; (**D**) final viscosity under different potassium treatments in 2019–2021; (**E**) setback under different potassium treatments in 2019–2021; (**F**) pasting temperature under different potassium treatments in 2019–2021.

The starch RVA profile is an important indicator for evaluating the eating quality of rice. A higher peak viscosity and breakdown, along with a lower setback, indicate better eating quality. With the delayed application of potassium fertilizer, the peak viscosity and breakdown in each treatment showed the highest values in K4 and the lowest in K1. The peak viscosity and breakdown in the potassium fertilizer delayed treatments were significantly increased, by 15.63~22.19% and 19.49~38.77%, respectively, compared to K1 (Figure 6A,C). The setback value was significantly reduced, by 18.56~39.20%, in the delayed potassium fertilizer treatments (Figure 6E). However, no significant differences in peak viscosity, breakdown, and setback were found between K2 and K3 treatments. With the delayed potassium fertilizer application, the hot viscosity and final viscosity showed an overall increasing trend (Figure 6B,D), while the gelatinization temperature showed a decreasing trend, although the differences between treatments were not significant (Figure 6F).

### 3.7. Taste Value

**Figure 7:**
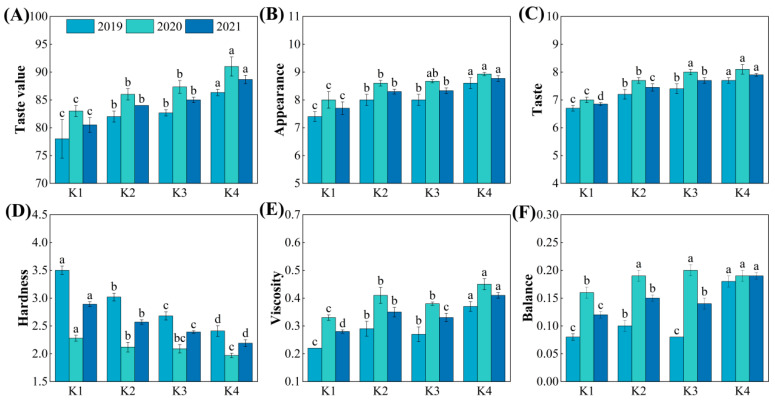
Effect of delayed potassium fertilizer application on rice taste value. Different lowercase letters indicate significant differences among treatments within the same year at *p* < 0.05. (**A**) Taste value under different potassium treatments in 2019–2021; (**B**) appearance under different potassium treatments in 2019–2021; (**C**) taste under different potassium treatments in 2019–2021; (**D**) hardness under different potassium treatments in 2019–2021; (**E**) viscosity under different potassium treatments in 2019–2021; (**F**) balance under different potassium treatments in 2019–2021.

The rice taste value is also an important indicator for evaluating the eating quality of rice. With the delayed application of potassium fertilizer, the taste value, appearance, taste, and viscosity of the rice showed an overall increasing trend, with K4 being the highest and K1 the lowest. The taste value, appearance, taste, and viscosity of the delayed potassium fertilizer treatments were significantly increased, by 4.35~10.14%, 7.79~13.85%, 8.76~15.33%, and 18.18~49.09%, respectively, compared to K1 (Figure 7A–C,E). However, no significant differences were observed between K2 and K3 treatments. With the delayed potassium fertilizer application, the hardness of rice showed a decreasing trend, with the delayed potassium fertilizer treatments significantly reducing hardness by 11.07~24.22% compared to K1 (Figure 7D). No significant differences were observed for balance and springiness between treatments (Figure 7F).

### 3.8. Processing and Appearance Quality

**Figure 8:**
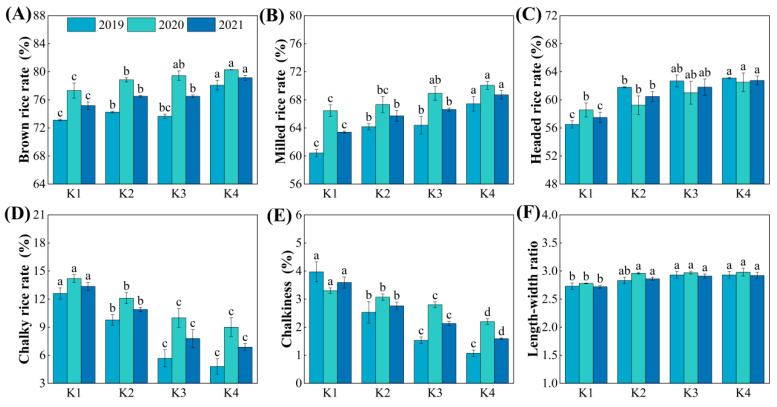
Effect of delayed potassium fertilizer application on rice processing and appearance quality. Different lowercase letters indicate significant differences among treatments within the same year at *p* < 0.05. (**A**) Brown rice rate under different potassium treatments in 2019–2021; (**B**) milled rice rate under different potassium treatments in 2019–2021; (**C**) head rice rate under different potassium treatments in 2019–2021; (**D**) chalky rice rate under different potassium treatments in 2019–2021; (**E**) chalkiness under different potassium treatments in 2019–2021; (**F**) length-to-width ratio under different potassium treatments in 2019–2021.

From a processing quality perspective, with the delayed application of potassium fertilizer, the brown rice rate, milled rice rate, and headed rice rate all showed an overall increasing trend, with K1 > K2 > K3 > K4. The delayed potassium fertilizer treatments significantly increased these rates by 1.77~5.28%, 3.64~8.39%, and 5.19~9.18%, respectively, compared to K1. However, no significant differences were observed between K2 and K3 treatments (Figure 8A–C).

From an appearance quality perspective, with the delayed application of potassium fertilizer, the chalky rice rate and chalkiness degree showed an overall decreasing trend, with K4 having the lowest values. The delayed potassium fertilizer treatments significantly reduced the chalky rice rate and chalkiness degree, by 18.43~48.56% and 23.02~55.25%, respectively, compared to K1 (Figure 8D,E). The length–width ratio showed an overall increasing trend; however, the differences between treatments were generally not significant (Figure 8F).

### 3.9. Yield

**Figure 9:**
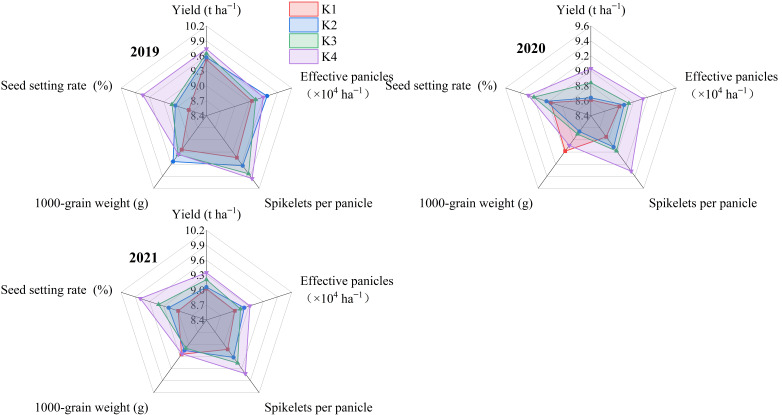
Effect of potassium fertilizer delayed application on rice yield. Statistical significance of yield and yield components among treatments was determined by ANOVA followed by LSD test (*p* < 0.05) based on original data; Figure 9 illustrates the relative performance of yield components.

With the delayed application of potassium fertilizer, the rice yield showed an increasing trend. The K3 and K4 treatments significantly increased yield, by 1.93% and 3.48%, respectively, compared to the K1 treatment, while no significant difference was observed between K2 and K1 treatments (Figure 9).

Regarding yield components, K3 showed a significant increase of 3.72% in the spikelets per panicle and 3.63% in the seed setting rate compared to K1, while K4 showed a significant increase of 6.57% and 7.01%, respectively. There were no significant differences in the effective panicles or 1000-grain weight between treatments.

### 3.10. Correlation Analysis

**Figure 10:**
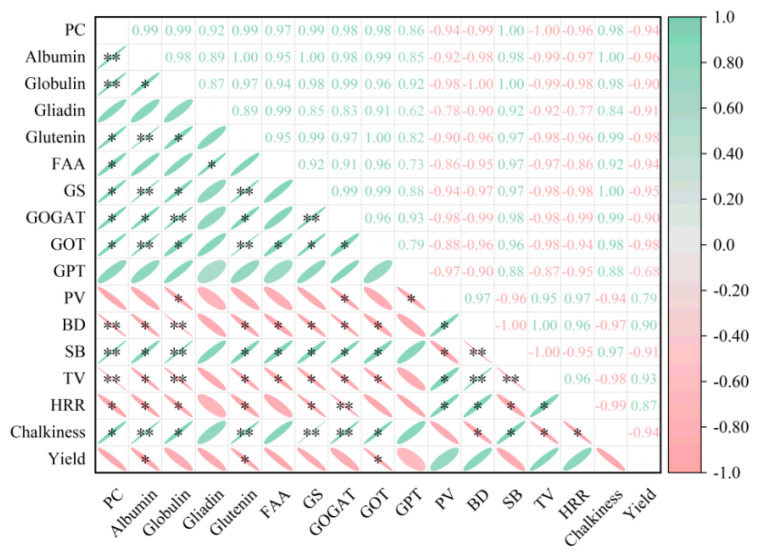
Correlation analysis of various indicators under potassium fertilizer management. * and ** indicates significant differences at 5% and 1% probability level respectively. PC: Relative protein content; FAA: Free amino acid; PV: Peak viscosity; BD: Breakdown; SB: Setback; TV: Taste value; HRR: Headed rice rate.

Correlation analysis showed that PC was significantly or highly positively correlated with albumin, globulin, glutenin, FAA, GS, GOGAT, GOT, SB, and chalkiness, while it was significantly or highly negatively correlated with BD, TV, and HRR (Figure 10). These results suggest that higher levels of storage proteins, especially gliadin and glutelin, may impair starch gelatinization and reduce palatability by increasing rice hardness and decreasing stickiness.

BD and TV were significantly positively correlated with HRR, while they were significantly or highly negatively correlated with PC, albumin, globulin, glutenin, FAA, GS, GOGAT, GOT, SB, and chalkiness, indicating that enhanced nitrogen assimilation may promote protein accumulation but concurrently reduce sensory quality.

## 4. Discussion

### 4.1. Response of Rice Protein Content to Delayed Potassium Fertilizer Application

Rice protein content is not only controlled by genetic factors but is also highly sensitive to environmental conditions [31]. Potassium, as a companion cation, can facilitate the absorption and translocation of nitrate ions, thereby enhancing protein and amino acid synthesis, as well as their transport [21]. In this study, we found that during the grain-filling process, the relative protein content in rice grains first decreased and then increased (Figure 1A–C). This pattern may be attributed to the mid-filling stage, which is a critical period for rapid starch synthesis and accumulation. The massive starch deposition diluted the proportion of protein in the grains, resulting in a “dilution effect,” indicating that carbon and nitrogen metabolism compete and coordinate with each other during grain-filling. Free amino acids, as precursors for protein synthesis, showed a declining trend throughout grain-filling (Figure 4), suggesting that they were continuously consumed for protein accumulation.

With the delayed application of potassium fertilizer, the contents of grain protein and its fractions (albumin, globulin, gliadin, and glutelin) decreased (Figure 1 and Figure 2), and their relative proportions also declined accordingly (Figure 3). This may be because, under late K application, carbon metabolism became more vigorous than nitrogen metabolism in the grains, significantly promoting starch accumulation. As a result, total dry matter increased, but the rate of protein synthesis did not rise proportionally, leading to a reduction in the relative protein content [32]. Notably, although delayed K application restricted protein accumulation, it markedly enhanced carbon metabolism, as evidenced by accelerated sucrose-to-starch conversion, faster grain-filling, reduced grain abortion, and a higher seed-setting rate, ultimately resulting in increased yield (Figure 9). This is similar to previous research. The backward shift of potassium fertilizer can effectively increase the leaf area and chlorophyll content of plants, promote the absorption and transfer of photosynthetic electrons, thereby improving the capture, absorption, and utilization of light energy by plants, enhancing the strong photosynthetic production capacity of leaves, and promoting the accumulation of dry matter and the formation of yield [33,34,35].

These findings suggest that delayed K application creates a trade-off between yield and quality: on the one hand, it improves grain-filling and yield; on the other hand, it lowers protein content, which may positively influence eating quality. Although delayed potassium application reduced the activities of key enzymes involved in protein synthesis and resulted in lower protein and free amino acid contents, this response should not be interpreted as a negative effect in the context of rice quality formation. In rice, excessive protein accumulation is well known to interfere with starch gelatinization, increase grain hardness, and ultimately impair eating quality. Therefore, the observed reduction in protein synthesis represents a compositional adjustment that favors starch-dominated endosperm structure rather than a physiological deficiency. Importantly, under the K4 treatment, although grain protein content was moderately reduced, the final protein level remained above 7%, which is generally considered sufficient to meet basic dietary protein requirements in rice-based diets. This indicates that the decrease in protein content does not necessarily compromise the nutritional value of rice. Instead, it reflects a quality-oriented optimization strategy that balances improved eating quality with adequate nutritional supply. In this study, such compositional rebalancing was accompanied by improved starch pasting properties, higher taste values, and a modest but significant yield increase, demonstrating that targeted regulation of protein synthesis through potassium management can enhance rice palatability without sacrificing its functional role as a staple food.

### 4.2. Response of Key Protein Synthesis Enzyme Activities in Rice to Delayed Potassium Fertilizer Application

The biosynthesis of proteins depends on the coordinated action of multiple key enzymes, among which glutamine synthetase (GS), glutamate synthase (GOGAT), and aminotransferases (GOT, GPT) are central components of the nitrogen metabolism pathway. These enzymes directly participate in ammonia assimilation and amino acid transformation, thereby regulating the dynamics of protein accumulation in rice grains [36,37]. In plants, potassium is mainly distributed as a soluble ion in the cytoplasm and vacuoles or adsorbed onto protoplasmic colloids. Due to its high membrane permeability, K^+^ can rapidly participate in the regulation of various enzyme activities [38]. Potassium enhances protein synthesis metabolism by activating key enzymes, increasing their substrate affinity, and stimulating membrane-bound enzymes, thus regulating protein biosynthesis [39].

In the present study, the available K content in the experimental field was at a deficient level (Table 1) [40]. As K fertilizer application was delayed, the activities of GS, GOGAT, GOT, and GPT in rice grains showed a decreasing trend (Figure 5). This suggests that during early grain-filling, insufficient potassium supply may weaken the synergistic relationship between potassium and nitrogen assimilation, thereby altering the K/N balance in assimilates transported to the grain. Potassium plays a key role in phloem transport and ionic regulation; thus, reduced early K availability can constrain nitrogen delivery and assimilation capacity, leading to lower activities of nitrogen-metabolizing enzymes. As potassium supply is shifted to later stages, sink strength for carbohydrate accumulation is enhanced, promoting starch deposition as the dominant metabolic priority during grain filling. This shift reduces the relative demand for nitrogen assimilation and protein synthesis (Figure 1D–F), resulting in decreased activities of GS, GOGAT, GOT, and GPT. In addition, potassium is known to directly influence enzyme activation and gene expression associated with nitrogen metabolism. Although gene expression was not measured in this study, previous evidence suggests that potassium deficiency can suppress nitrogen-assimilation pathways at both the enzymatic and transcriptional levels. Together, these mechanisms explain why delayed potassium application restricts nitrogen assimilation while favoring starch accumulation, ultimately improving eating quality, despite reduced protein synthesis.

### 4.3. Response of Rice Quality to Delayed Potassium Fertilizer Application and Its Relationship with Protein Content

Eating quality is the most important trait of rice; its evaluation encompasses multiple parameters. The starch RVA profile and taste value are commonly used as standard indicators [41,42]. Previous studies have shown that appropriate K fertilization can increase the paste consistency, peak viscosity, and hot paste viscosity of rice flour, thereby improving eating quality. It can also enhance the milled rice rate and head rice rate, while reducing chalky grain rate and chalkiness [43]. Further research has indicated that increasing the proportion of panicle fertilizer could achieve even greater improvements in processing and appearance quality [44]. The findings of this study are largely consistent with these reports. With delayed K application, peak viscosity and breakdown increased, setback decreased, and the eating quality score improved, indicating a tendency toward better cooking and eating quality (Figure 6 and Figure 7). At the same time, delayed K application also increased the head rice rate and reduced the chalky grain rate and chalkiness, which helped to improve both processing and appearance quality (Figure 8).

The relationship between eating quality and protein content or protein fractions in rice remains controversial. Most studies have reported that increased protein content exerts a negative impact on eating quality; with higher protein levels, rice hardness increases, while stickiness, elasticity, and appearance decline, ultimately leading to reduced palatability [7,45]. Other studies, however, have suggested that breeding rice varieties with moderately low protein content can improve eating quality, whereas excessively high or low protein levels may both impair quality to varying degrees [46], indicating that the relationship between protein content and cooking quality may not be strictly linear. In addition, protein fractions with different solubility and structural properties have also been proposed as important factors contributing to differences in eating quality. For example, albumin has been reported to negatively affect eating quality, whereas globulin shows an opposite effect. Gliadin exhibits the strongest negative correlation with eating quality, followed by glutelin, although the latter remains the dominant component due to its relatively high abundance [47]. Moreover, some studies demonstrated that gliadin, compared with other protein fractions, markedly reduced rice viscosity and exerted a strong negative effect on eating quality, while also being significantly negatively correlated with rice elasticity and grain integrity [48].

This study found that rice breakdown and taste value were significantly or highly significantly negatively correlated with PC, albumin, globulin, glutelin, and FAA (Figure 10). A possible explanation is that rice with higher protein content has a denser internal structure, with smaller gaps between starch granules in the endosperm, which restricts starch gelatinization during cooking, leading to a higher gelatinization temperature and reduced rice softness [49]. Overall, delayed potassium application reduced the contents of protein and its fractions, thereby alleviating the restrictive effect of protein on starch gelatinization, improving eating quality, while also enhancing processing and appearance quality, thus helping to meet consumer demand for high-quality rice.

It should be noted that the applicability of the present findings should be interpreted in the context of both rice genotype and soil potassium status. This study was conducted using a hybrid indica cultivar grown on potassium-deficient soil, where delayed potassium application effectively improved eating quality by reducing protein accumulation and enhancing starch functionality. Although similar trends may occur in other rice types, such as japonica or aromatic rice, differences in genetic background, grain composition, and sink strength may lead to distinct responses to potassium timing. In addition, soil potassium availability is a critical factor determining fertilizer response. In soils with moderate to high available K, the benefits of delayed potassium application may be less pronounced; the optimal basal-to-panicle K ratio may differ from the 4:6 ratio identified here. Therefore, the optimal potassium split strategy should be considered as variety- and site-specific. Further multi-variety and multi-site studies across contrasting soil potassium levels are needed to validate and refine potassium management strategies for broader application. The experiment was conducted over three consecutive growing seasons, during which climatic conditions showed no pronounced differences and were generally representative of local rice production. Accordingly, the use of three-year mean values helped to minimize year-to-year climatic variability and strengthened the robustness of treatment comparisons. It should also be acknowledged that soil nutrient dynamics were not monitored during the growing season in this study. Although the experimental soil was characterized as potassium-deficient prior to transplanting, soil-available potassium levels at different growth stages were not measured. Therefore, the proposed explanation that delayed potassium application may reduce potassium fixation and improve plant availability is based on established soil potassium dynamics reported in previous studies, rather than direct measurements from the present experiment. The lack of in-season soil nutrient data limits the ability to quantitatively assess soil potassium status (e.g., low, medium, or high availability) during crop growth. Future studies incorporating dynamic measurements of soil potassium and other nutrients would provide a more comprehensive understanding of soil–plant potassium interactions under delayed potassium fertilization.

It should also be acknowledged that carbon metabolism-related parameters were not directly measured in this study. Instead, the proposed coordination between carbon and nitrogen metabolism was inferred from changes in grain composition (protein fractions and free amino acids) and starch functional properties (RVA profiles and eating quality), which are widely recognized as integrative outcomes of carbon–nitrogen allocation during grain filling. While this indirect evidence supports the proposed mechanism, further studies incorporating direct measurements of carbon metabolism and assimilate partitioning would help to more comprehensively elucidate the physiological basis of potassium timing effects. In addition, an important methodological limitation of this study should be explicitly acknowledged. Although the total potassium fertilizer input was kept constant among treatments, potassium was supplied exclusively in the form of potassium chloride (KCl); the experiment manipulated only the timing of KCl application rather than the chemical form of potassium. Consequently, the altered application timing affected the temporal availability of both K^+^ and Cl^−^ simultaneously. While the identical total KCl dose among treatments minimizes differences in overall chloride input, it does not exclude the possibility that split application modified the temporal availability of chloride during crop growth. Therefore, it is not methodologically possible to attribute the observed effects exclusively to potassium without additional treatments using chloride-free potassium fertilizers (e.g., K_2_SO_4_) or compensatory chloride controls. The present results should thus be interpreted as responses to KCl application timing rather than to potassium alone. Future studies incorporating alternative potassium sources or explicit chloride controls are necessary to disentangle the individual contributions of K^+^ and Cl^−^ and to more precisely clarify their respective roles in regulating grain composition, yield, and quality.

## 5. Conclusions

With the delayed application of potassium fertilizer, the activities of the enzymes GS, GOGAT, GOT, and GPT in the grains decrease, leading to a reduction in the content of protein, albumin, globulin, gliadin, glutenin, and free amino acids. However, the peak viscosity, breakdown, and taste value increase, while the setback decreases, thereby improving the eating quality of rice. In addition, with the delayed application of potassium fertilizer, the brown rice rate, milled rice rate, headed rice rate, and yield gradually increase, while the chalky rice rate and chalkiness decrease, which benefits the optimization of processing and appearance quality. Overall, the K4 treatment is beneficial for synergistically improving both yield and eating quality. These findings highlight the pivotal role of potassium fertilizer management in regulating carbon–nitrogen metabolism and quality formation in rice, providing important physiological insights and practical guidance for developing cultivation strategies aimed at producing high-yield, high-quality rice to meet consumer demands.

## Figures and Tables

**Table 1:** Basic soil chemical properties.

Year	pH	Organic Matter (g kg^−1^)	Total N (g kg^−1^)	Available N (mg kg^−1^)	Available P (mg kg^−1^)	Available K (mg kg^−1^)
2019	6.85	27.18	1.81	97.58	28.30	57.42
2020	6.55	29.05	1.64	86.45	24.19	50.13
2021	6.62	29.10	1.72	88.28	26.24	54.26

## Data Availability

The data presented in this study are available on request from the corresponding author. The data are not publicly available due to privacy and ethical restrictions.
